# Structure and function of persulfide dioxygenase from *Pseudomonas aeruginosa*: Implications on H_2_S homeostasis and interplay with nitric oxide

**DOI:** 10.1016/j.isci.2025.114586

**Published:** 2025-12-30

**Authors:** Francesca Giordano, Francesca Troilo, Martina Roberta Nastasi, Lorenzo Caruso, Marta Mellini, Carlo Travaglini-Allocatelli, Giorgio Giardina, João B. Vicente, Giordano Rampioni, Adele Di Matteo, Elena Forte, Alessandro Giuffrè

**Affiliations:** 1 Department of Biochemical Sciences “A. Rossi Fanelli”, Sapienza University of Rome, 00185 Rome, Italy; 2 Institute of Molecular Biology and Pathology, National Research Council, 00185 Rome, Italy; 3 Department of Science, Roma Tre University, 00146 Rome, Italy; 4 Instituto de Tecnologia Química e Biológica António Xavier, Universidade Nova de Lisboa, Oeiras, Portugal; 5 IRCCS Fondazione Santa Lucia, Rome, Italy

**Keywords:** biochemistry, microbiology, structural biology

## Abstract

Hydrogen sulfide is an important signaling molecule, beneficial at physiological concentrations but harmful at higher levels, due to which a tight control of its bioavailability is essential. Here, we investigated persulfide dioxygenase, an enzyme involved in H_2_S catabolism, from the pathogen *Pseudomonas aeruginosa* (*Pa*PDO). Deletion of the gene *pdo* led to a 4-fold increase in H_2_S concentration, confirming its physiological role. The recombinant enzyme was structurally characterized at 2.06 Å resolution and assigned to the metallo-β-lactamase superfamily. Compared with its human homolog, *Pa*PDO displayed a different dimerization area and a larger active site, suggesting different substrate preferences. Functionally, *Pa*PDO catalyzed glutathione persulfide dioxygenation with a high turnover rate, and its activity was enhanced by reduced glutathione. Interestingly, the results show that *Pa*PDO binds to nitric oxide, which reversibly inhibits its catalytic activity. These findings reveal a novel mechanism of crosstalk between hydrogen sulfide and nitric oxide signaling and provide insights into redox regulation in a multidrug-resistant pathogen.

## Highlights

• *Pa*PDO 3D structure displays the typical metallo-β-lactamase fold with a dimeric organization
• Deletion of the gene *pdo* leads to a 4-fold increase in hydrogen sulfide concentration
• *Pa*PDO catalyzes glutathione persulfide dioxygenation with a high turnover rate
• Nitric oxide binds to *Pa*PDO, potently and reversibly inhibiting its activity

## Graphical abstract

**Figure:**
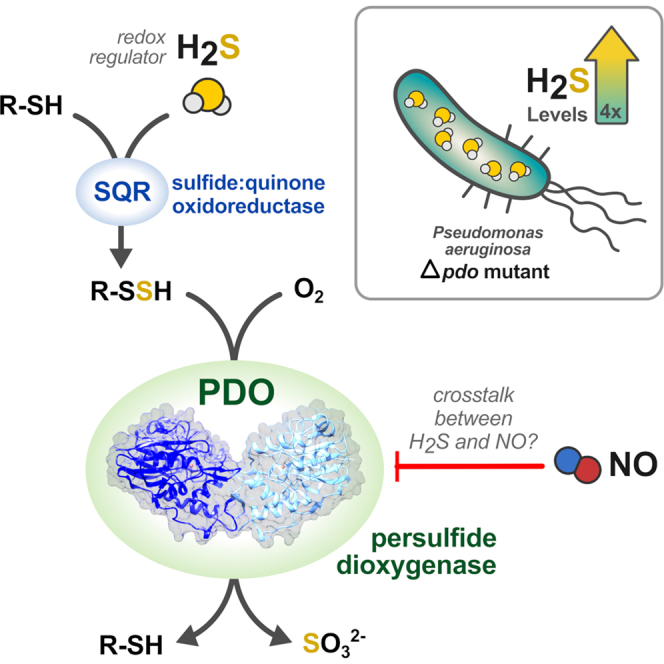

## Introduction

Hydrogen sulfide (H_2_S) is a pleiotropic gaseous signaling molecule alongside nitric oxide (NO) and carbon monoxide.^1^^,^^2^^,^^3^ While a pivotal physiological role for H_2_S has been recognized in multi-cellular organisms, the impact of this molecule on bacterial physiology and pathophysiology remains largely unexplored and sometimes controversial. Of note, H_2_S and related sulfane sulfur-containing species were found to promote virulence in some bacteria.^4^^,^^5^^,^^6^ While a number of studies have underscored the significance of H_2_S in protecting bacteria from antibiotics and oxidative stress,^7^^,^^8^^,^^9^^,^^10^ others have raised doubts about the universality of these functions among bacteria.^5^^,^^11^^,^^12^^,^^13^

To maintain physiological H_2_S levels within a nontoxic range, a balance between its production and breakdown is essential. In bacteria, H_2_S synthesis can vary depending on species and growth conditions and, except for sulfate-reducing bacteria, this process is largely mediated by cystathionine β-synthase (CBS), cystathionine γ-lyase (CSE), and 3-mercaptopyruvate sulfurtransferase.^14^ H_2_S breakdown occurs through the catabolic pathway, in which H_2_S is oxidized to thiosulfate and sulfate in the presence of O_2_. The H_2_S-oxidizing unit comprises sulfide:quinone oxidoreductase (SQR), persulfide dioxygenase (PDO), rhodanese, and sulfite oxidase (SO).^15^

PDO catalyzes the conversion of glutathione persulfide (GSSH) to reduced glutathione (GSH) and sulfite (SO_3_^2−^), according to the following equation:

(Equation 1) $$\text{GSSH}+O_{2}+H_{2}O\;\to \;\text{GSH}+{\text{SO}}_{3}^{2-}+2H^{+}$$

The most extensively characterized PDO is its human homolog, hPDO, also known as ethylmalonic encephalopathy protein 1 (ETHE1), which plays a crucial role in mitochondrial sulfide catabolism.^16^^,^^17^^,^^18^ PDOs have been found in many organisms, including plants^19^^,^^20^ and bacteria.^21^^,^^22^^,^^23^ In some bacteria, the gene *pdo* is fused to other sulfur metabolism-related genes.^21^^,^^24^^,^^25^ Most PDOs preferentially use GSSH as the substrate,^16^^,^^18^ even though hPDO can also use glutathione polysulfide as a substrate.^26^

PDOs have been categorized into the following three types: PDO I, commonly found in animals, plants, and some bacteria; PDO II, predominantly present in proteobacteria; and PDO III, found in both bacteria and archaea.^15^^,^^21^^,^^22^^,^^23^ They all belong to the metallo-β-lactamase (MBL) superfamily, which comprises proteins with diverse functions characterized by an αβ/βα sandwich fold wherein the active site is located at the interface of the two αβ-modules.^27^^,^^28^ The 3D structures of various PDOs of different subtypes and origins, including those from humans (hPDO, PDB: 4CHL),^29^ *Arabidopsis thaliana* (*At*ETHE1, PDB: 2CGU),^19^ and bacteria such as *Myxococcus xanthus (Mx*PDO, PDB: 4YSB)^22^ and *Pseudomonas putida* (*Pp*PDO, PDB: 4YSK, 4YSL), have been resolved.^22^

The Gram-negative opportunistic pathogen *Pseudomonas aeruginosa* (*Pa*) is a common cause of acute lung, soft tissue, and systemic infections, particularly in immunocompromised hosts, as well as of hard-to-eradicate chronic pulmonary infections in individuals with cystic fibrosis.^30^^,^^31^ Infections caused by *Pa* were associated with more than 500,000 deaths in 2019, and antimicrobial resistance of this pathogen is increasing worldwide, calling for the development of new anti-*Pa* therapeutic strategies.^32^^,^^33^ In this context, studies showing that H_2_S decreases antibiotics efficacy and controls the expression of virulence factors in *Pa*^6^^,^^7^^,^^9^ have highlighted the enzymes involved in H_2_S metabolism as possible targets for the development of new drugs that can affect resistance and pathogenicity in this bacterium. Despite recent findings reporting that H_2_S does not affect antibiotic resistance in *Pa*, at least in some strains and environmental conditions,^12^ this pathogen possesses all the genes putatively encoding the enzymes involved in both H_2_S synthesis and catabolism,^15^ suggesting that a fine control of H_2_S levels could be pivotal for *Pa* pathophysiology.

Herein, we present a thorough characterization of the PDO from *Pa* (*Pa*PDO) through a multidisciplinary approach, including microbiological, structural, and biochemical analyses. Our results show that *Pa*PDO is involved in H_2_S catabolism in *Pa*, with *pdo* deletion resulting in increased H_2_S levels compared with those in the parental strain. The protein 3D structure displays the typical MBL fold with a dimeric organization characterized by a large contact surface between monomers. Enzyme kinetics determined by high-resolution respirometry revealed that *Pa*PDO has GSSH dioxygenase activity, which was proven to be inhibited by NO, unveiling a yet undescribed putative crosstalk mechanism between NO and H_2_S.

## Results

### *pdo* is involved in H_2_S breakdown in *Pa*

**Figure 1:**
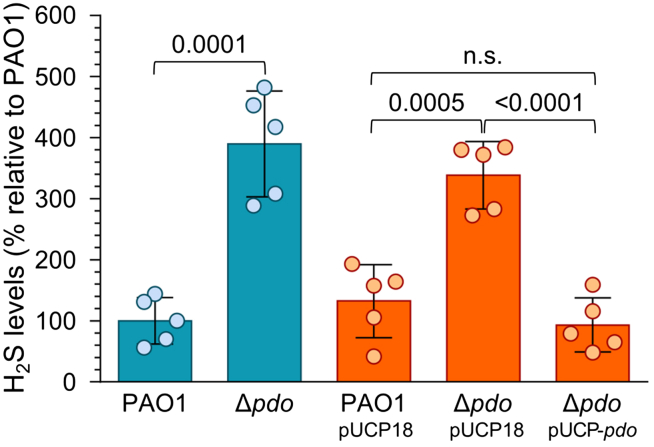
Role of the gene *pdo* in H_2_S catabolism in *Pa* H_2_S levels detected with PAO1, its isogenic Δ*pdo* mutant (blue bars), and the same strains carrying either the pUCP18 empty vector or the pUCP18-derived plasmid pUCP-*pdo* for constitutive *pdo* expression (orange bars). Data are reported as the percentage relative to PAO1. The mean and standard deviations were obtained from five independent experiments. *p* values are indicated.

To investigate the role of the gene *pdo* in H_2_S catabolism, we generated a Δ*pdo* markerless deletion mutant in PAO1 and a plasmid for *pdo* ectopic expression (Figure S1). We then compared the H_2_S levels present in the cultures of PAO1 and the isogenic Δ*pdo* mutant by using the lead acetate detection method.^34^ Deletion of *pdo* did not affect *Pa* growth kinetics (Figure S2); however, it resulted in ca. 4-fold higher H_2_S levels compared with those in PAO1. Notably, wild-type H_2_S levels were restored in the Δ*pdo*(pUCP-*pdo*) complemented strain (Figure 1). These data demonstrate that *Pa*PDO is involved in regulating H_2_S levels in *Pa*.

### Biochemical properties of purified *Pa*PDO

**Figure 2:**
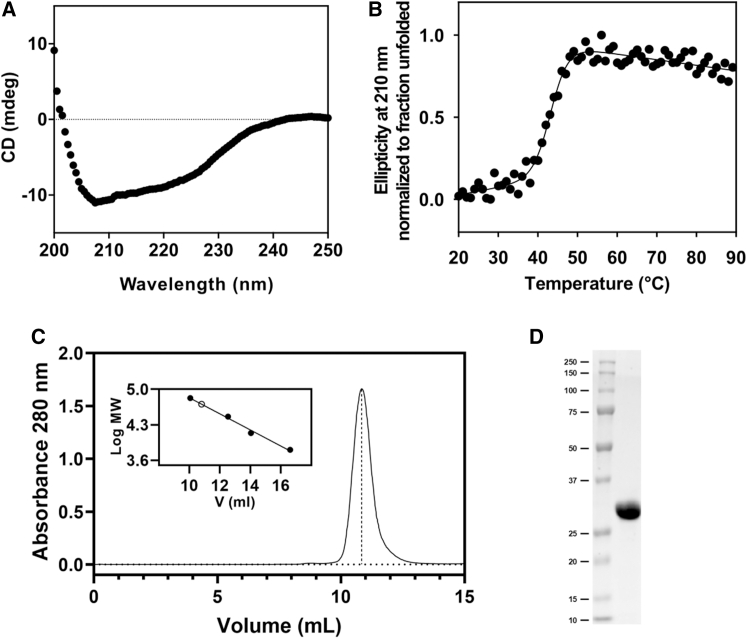
Recombinant *Pa*PDO is correctly folded and displays dimeric assembly in solution (A) Far-UV CD spectra collected at 20°C. The spectrum displays the typical features of a mixed α/β secondary structure content (15% α and 23% β, as calculated by BeStSel webserver [https://bestsel.elte.hu/index.php]). (B) Thermal denaturation profile followed by monitoring the CD signal at 210 nm at increasing temperature; curve fitting yielded an apparent melting temperature (*T*_m_) of 43.3°C. (C) SEC analysis. Based on the calibration curve shown in the inset (full circles: protein markers; empty circle: *Pa*PDO), according to the observed elution volume (10.8 mL), *Pa*PDO has an MW of 52 kDa, consistent with the dimeric assembly of the protein in solution. (D) SDS-PAGE of the purified *Pa*PDO.

To characterize *Pa*PDO, we produced the recombinant protein in *Escherichia* *coli* (*E. coli*), with a yield of approximately 40 mg/L of culture. By far-UV circular dichroism (CD) spectroscopy, the purified enzyme was shown to display the typical features of a mixed α/β secondary structure (Figure 2A) and a cooperative irreversible thermal denaturation profile (Figure 2B), indicating correct folding.

By size-exclusion chromatography (SEC) analysis, *Pa*PDO was found to adopt a dimeric assembly in solution (Figure 2C), as previously reported for *Pp*PDO,^22^ and it was purified to homogeneity (Figure 2D). Metal content of the purified protein used for the biochemical assays was determined using the ferrozine assay (Figure S3), which indicated incomplete iron loading (approximately 0.7 mol Fe/mol *Pa*PDO), in analogy to other recombinant PDOs of bacterial or animal origin.^18^^,^^21^^,^^35^

### *Pa*PDO structural characterization

**Figure 3:**
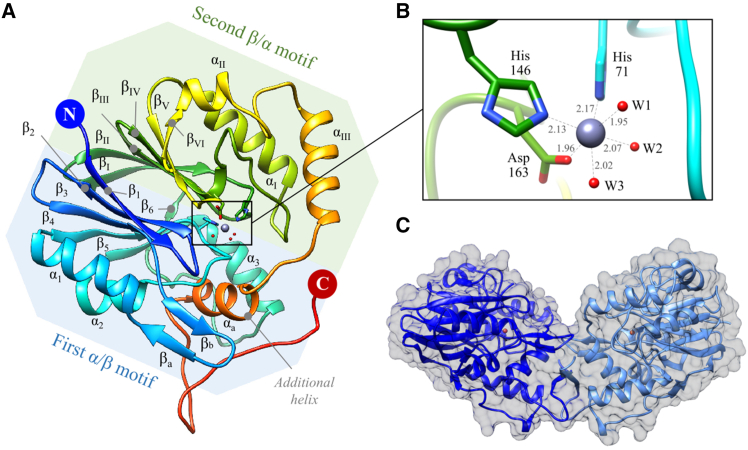
*Pa*PDO structure (A) Rainbow ribbon representation of the structure of one subunit of the *Pa*PDO dimer, with the residues involved in metal (large sphere) coordination (His71, His146, and Asp163) represented in sticks, and the three metal coordinating waters represented in red spheres. The two α/β motifs are highlighted in blue and green, respectively. The first motif comprises eight β-strands and three α-helices organized as β_1_↑β_2_↓β_3_↑α_1_β_4_↑α_2_β_5_↑α_3_β_6_↑ with a two-stranded β-sheet (β_a_↑β_b_↓) connecting β_3_ to α_1_. The second motif consists of six β-strands and three α-helices organized as β_Ι_↑β_ΙΙ_↓β_ΙΙΙ_↑β_ΙV_↑α_Ι_β_V_↑β_VΙ_↑α_ΙΙ_α_ΙΙΙ_. A lengthy loop connects α_ΙΙΙ_ to an additional α_a_-helix, which folds back and intercalates between the helices α_2_ and α_3_ of the first motif. (B) Close view of the metal-binding site. (C) Dimeric organization of *Pa*PDO.

The *Pa*PDO 3D structure was solved by X-ray crystallography at 2.06 Å in the P3_1_21 space group. Statistics for data collection and refinement are reported in Table S1. *Pa*PDO has the characteristic MBL fold,^28^ consisting of an αβ/βα sandwich with two central β-sheets stacked together and surrounded by α-helices (Figure 3A). The active site is located at the αβ/βα motif interface, where residues of both βα motifs participate in the coordination of the metal ion. In the active site, we found a single metal ion coordinated by His71, His146, Asp163, and three water molecules (W1, W2, and W3 in Figure 3B) in an octahedral coordination geometry, known as a 2-His1-carboxylate facial triad.^22^^,^^36^ The X-ray fluorescence spectrum and anomalous signal analysis of *Pa*PDO crystals indicated a mixed metal occupancy with an abundance of Zn and Ca ions, rather than Fe ions (Figure S4). Likely, these metals have replaced Fe during the purification and/or crystallization process, a common finding for non-heme Fe-binding proteins.^37^^,^^38^^,^^39^

**Figure 4:**
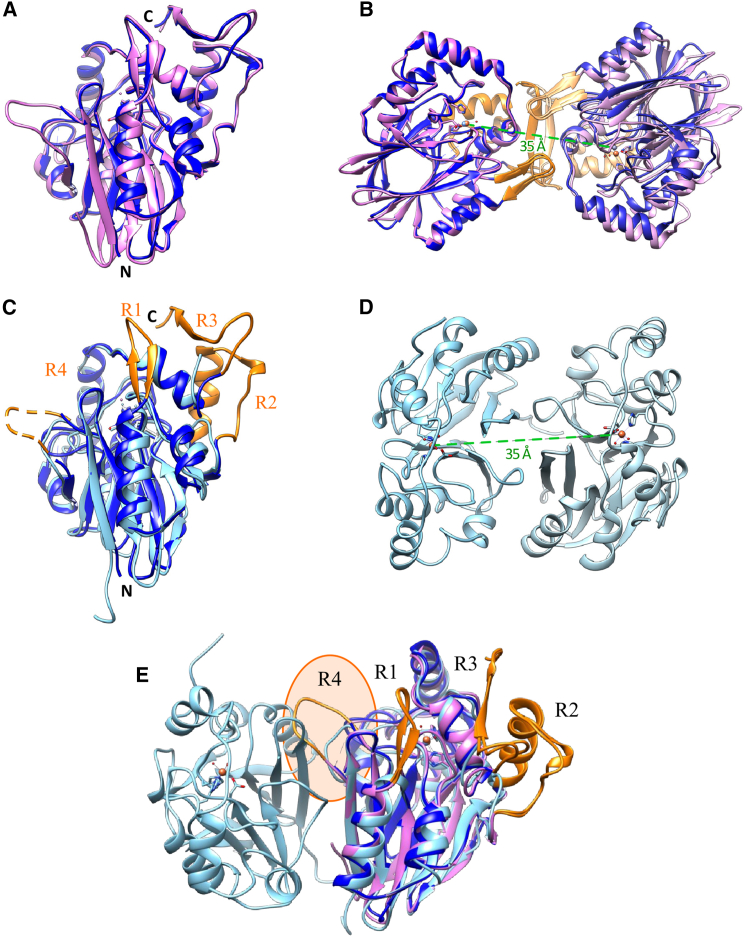
Structural comparison (A and B) Structural superposition of *Pa*PDO (blue) and *Pp*PDO (PDB: 4YSK, magenta) as single chains (A) and homodimers (B). (C) Structural superposition of *Pa*PDO (blue) and hPDO (PDB: 4CHL, light blue) as single chains; Region 4 could not be fitted in the electron density map and thus was modeled based on *Pp*PDO and is represented as a dotted line in the figure. (D) hPDO homodimer. (E) Ribbon representation of the *Pa*PDO (blue) and *Pp*PDO (magenta) single chains superimposed to the hPDO homodimer (PDB: 4CHL, light blue). Regions 1–4 are represented in orange.

*Pa*PDO crystallized as a homodimer, consistent with the protein’s physiological assembly in solution assessed by SEC (Figure 2C). In the dimer, the two monomers were found to be organized in a “butterfly wing” arrangement in which the C-terminal region of one monomer forms a two-stranded β-sheet with the other subunit (Figure 3C). The dimerization interface area was found to be quite large, with a buried surface area of 1255 Å^2^, corresponding to a dissociation energy of 12.9 kcal/mol according to PISA (Proteins, Interfaces, Structures and Assemblies) analysis.^40^ Sequence alignment and structural superposition of *Pa*PDO with *Pp*PDO (PDB: 4YSK)^22^ revealed high similarity in terms of sequence (60.6% identity) and structural organization (rmsd across all 279 pairs: 0.857 Å) (Figures 4A–4C and S5), while significant differences were observed when comparing the single chain of *Pa*PDO to the human homolog h*PDO* (PDB: 4CHL;^29^ rmsd across all 226 pairs: 2.069 Å, sequence identity: 24.5%).

Some regions of *Pa*PDO conserved in *Pp*PDO were not found in hPDO, particularly (1) region 1 (R1, res: 37–48) containing the two-stranded β_a_β_b_ sheet connecting β_3_ to α_1_; (2) region 2 (R2, res: 98–120), connecting α_3_ to β_6_; (3) a long C-terminal extension (R3), which extends beyond the core structure of the protein; and (4) a six-residue insertion in the loop connecting β_V_ to β_VI_ (R4, res: 208–215) (Figures 4C and S5). Importantly, R1–R3 mediate dimeric organization in *Pa*PDO (Figures 4B and S5). Consistently, the dimer observed for hPDO was quite different (Figure 4D), with a smaller interface area (827 Å^2^) and a lower dissociation energy (8.1 kcal/mol) according to PISA analysis.^41^ Notably, while R4 is solvent-exposed in bacterial PDO dimers, in hPDO, it is part of the dimeric interface. This difference prevents bacterial PDOs from adopting the same quaternary structure as hPDO (Figure 4E). Additionally, the residues indicated as responsible for GSH binding in *Pp*PDO, including the three arginine residues electrostatically interacting with GSH (Arg174, Arg244, and Arg247),^22^ are conserved in *Pa*PDO, but some of them are not conserved in hPDO and other type I PDOs (Figure S6).

### *Pa*PDO has glutathione persulfide dioxygenase activity

**Figure 5:**
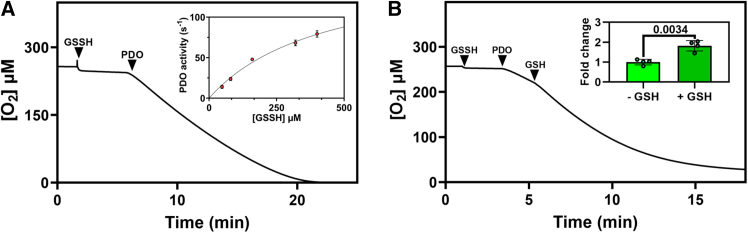
Kinetic analysis of *Pa*PDO activity (A) Representative oxygraphic trace representing O_2_ consumption (black line) by *Pa*PDO (5.3 nM holoprotein) in the presence of GSSH (320 μM). Inset: dependence of the reaction velocity on GSSH concentration (50–400 μM); each point represents the average of four independent experiments. (B) Effect of GSH (1 mM) on *Pa*PDO activity in the presence of 160 μM GSSH. Addition of GSH resulted in approximately 2-fold increase in *Pa*PDO activity. Inset: fold change in *Pa*PDO activity in the presence of GSH. The average of four independent experiments is reported together with the standard deviation. *p* value is indicated.

The dioxygenase activity of the isolated *Pa*PDO was assessed by high-resolution respirometry, which measures the O_2_ consumed upon the conversion of GSSH to GSH and SO_3_^2−^ in real time. Possible substrates other than GSSH, such as cysteine persulfide (CysSSH), GSH, or Na_2_S, were also tested, which revealed no detectable activity (data not shown). As shown in Figure 5A, in the presence of GSSH, the addition of the recombinant protein resulted in O_2_ consumption. Analysis of the reaction velocity dependence on [GSSH] revealed an estimated *K*_m_ higher than 400 μM and a *k*_*cat*_ of 165 s^−1^ (inset to Figure 5A). The *Pa*PDO catalytic activity exhibited nonlinear kinetics likely due to the low substrate affinity and/or protein auto-inactivation promoted by glutathionylation of cysteine residue(s) during turnover. According to Equation 2 (see STAR Methods), GSSH preparations contain equimolar GSH. Thus, although GSH itself does not act as a *Pa*PDO substrate, we investigated whether it could somehow affect PDO activity. At high [GSH] of 1 mM, we observed approximately 2-fold increase in the activity compared with that measured in the presence of GSSH (160 μM) alone (Figure 5). The same stimulatory effect was reported for hPDO,^18^ although its cause is still debated.^16^ Additionally, we tested *Pa*PDO activity at varied O_2_ concentrations and a fixed GSSH concentration (160 μM) and observed that *Pa*PDO has a high affinity for O_2_ (apparent *K*_*m*_ ≤ 7.4 μM, Figure S7).

### NO inhibits the persulfide dioxygenase activity of *Pa*PDO

**Figure 6:**
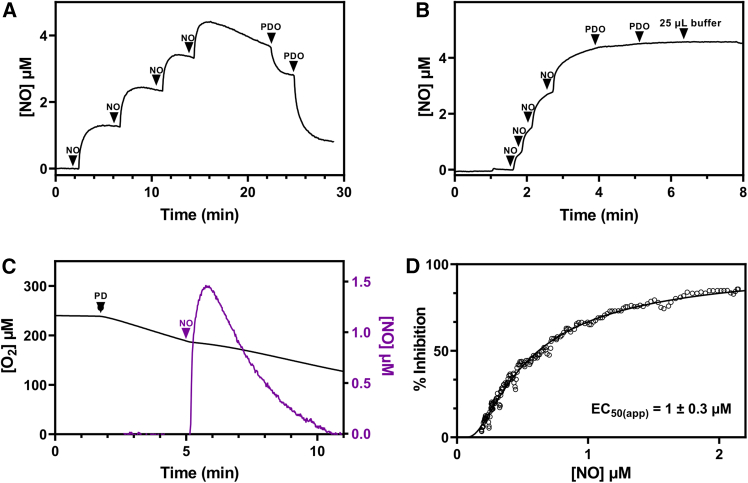
Interaction of *P*aPDO with NO (A) Ferrous *Pa*PDO binds to NO. Representative NO amperometric trace showing four consecutive additions of 1.1 μM NO to degassed buffer (100 mM sodium phosphate, pH 7.4, containing 1 U/mL ascorbic oxidase and 5 mM ascorbate to scavenge residual O_2_), followed by the addition of 0.5 μM and 1 μM degassed *Pa*PDO, previously incubated with 10-fold excess of GSSH. NO binding is evidenced as a decrease in amperometric signal, consistent with a stoichiometry of 1.1 ± 0.3 mol of NO per mol of holo *Pa*PDO (*n* = 4). (B) “As-prepared” untreated *Pa*PDO does not bind to NO. After four 1.1 μM NO additions to degassed buffer, no NO consumption was observed after the addition of two aliquots of 0.5 μM degassed *Pa*PDO in its ferric form (i.e., not preincubated with GSSH). (C) *Pa*PDO is reversibly inhibited by NO. Representative oxygraphic trace showing that the addition of 1.5 μM NO (purple line) to *Pa*PDO in turnover with GSSH results in enzyme inhibition (black line), activity being fully recovered upon NO exhaustion. (D) Percentage of *Pa*PDO inhibition plotted as a function of [NO], yielding an apparent *EC*_50_ value of 1.0 ± 0.3 μM NO. The average of eight independent experiments is reported together with the standard deviation.

The ability of NO to bind to *Pa*PDO was assessed amperometrically under anaerobic conditions by using an NO-selective electrode. Following the addition of *Pa*PDO pre-reduced with a 10-fold excess of GSSH to an NO solution, a fast decrease in NO concentration was observed (Figure 6A). The addition of the same amount of GSSH in the absence of the protein caused a much less pronounced decrease in the NO levels (Figure S8). A binding stoichiometry of 1.1 ± 0.3 mol of NO per mol of *Pa*PDO could be estimated. In contrast, NO binding to the “as prepared” (GSSH-untreated) *Pa*PDO was negligible (Figure 6B). Using the ferrozine assay, we found that iron was partially reduced in the “as prepared” untreated *Pa*PDO and fully reduced in GSSH-treated enzyme (Figure S3B). These data suggest that GSSH, when bound to the *Pa*PDO active site, facilitates not only full reduction of the non-heme iron but also complexation of NO to it, possibly by stabilizing the resulting nitrosyl Fe adduct. This is consistent with the proposed reaction mechanism for hPDO,^16^ in which persulfide binding to the active site prompts the non-heme ferrous iron toward O_2_ binding.

We then tested the effect of NO on the protein’s PDO activity. As evidenced in Figure 6C, upon the addition of NO to *Pa*PDO in turnover with GSSH, the rate of O_2_ consumption decreased due to the inhibition of *Pa*PDO. Inhibition was found to be reversible, as full activity recovery was observed (according to the nonlinear time course), once NO was completely consumed upon reaction with O_2_ in the solution (purple trace in Figure 6C). Consistently, NO inhibition of *Pa*PDO was more effective and persistent at lower O_2_ concentration (Figure S9). By plotting the percentage of inhibition as a function of NO concentration (Figure 6D), the apparent *EC*_50_ was estimated to be 1.0 ± 0.3 μM NO.

**Figure 7:**
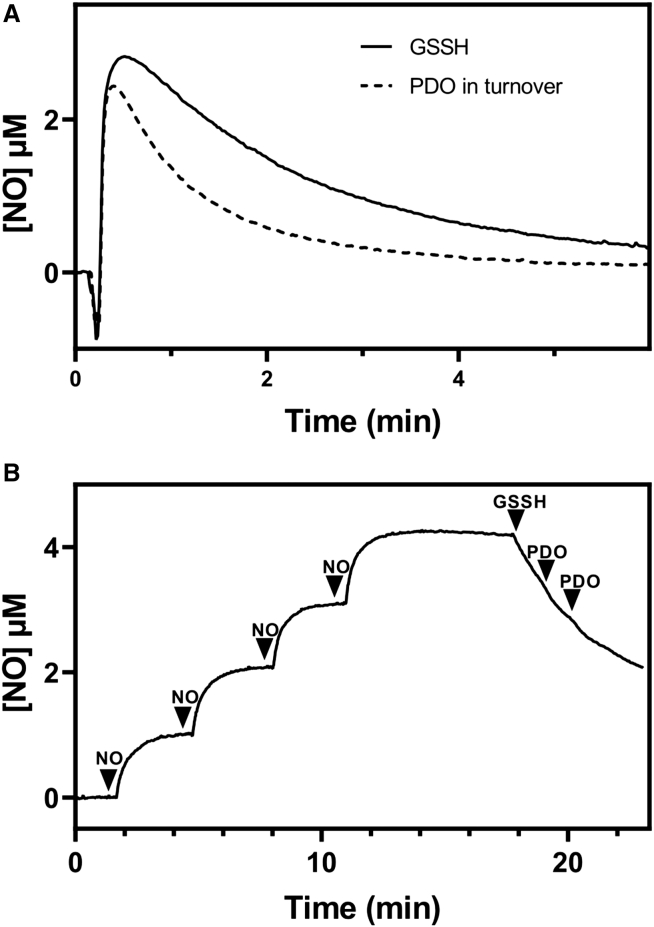
NO metabolism by *Pa*PDO (A) *Pa*PDO NO dioxygenase activity. Amperometric NO traces collected in the presence of *Pa*PDO in turnover with 160 μM GSSH (dashed black line) or GSSH alone at the same concentration as control (solid black line). In both cases, 2.2 μM NO was added at 200 μM O_2_. (B) Lack of NO reductase activity. Four aliquots of 1.1 μM NO were added to degassed buffer (100 mM sodium phosphate, pH 7.4, supplemented with 1 U/mL ascorbic oxidase and 5 mM ascorbate) to maintain anaerobic conditions. Subsequent addition of 80 μM GSSH led to NO consumption, but no change in the NO consumption rate was observed upon the addition of 5.3 nM *Pa*PDO holoenzyme.

Then, we tested whether *Pa*PDO has either NO dioxygenase or NO reductase activity. As for the former, we measured by NO amperometry the change in NO concentration in the presence of O_2_ and either *Pa*PDO in turnover with GSSH (160 μM) or GSSH alone at the same concentration as a control. As evidenced in Figure 7A, the rate of NO consumption in the presence of *Pa*PDO in turnover (dashed line) was higher than that observed with GSSH only (solid line). This result demonstrated that *Pa*PDO is endowed with NO dioxygenase activity, with an estimated turnover number of 2.4 ± 0.6 s^−1^ at 2.2 μM NO. On the contrary, *Pa*PDO is devoid of NO reductase activity. Indeed, as assessed under anaerobic conditions, although 80 μM GSSH per se reacts with NO, in line with previous reports,^42^ subsequent protein addition did not change the NO consumption rate (Figure 7B).

## Discussion

H_2_S is a signaling molecule involved in the regulation of a variety of cellular processes. Acting as a dual-faced player, it is beneficial at physiological concentrations but harmful at higher concentrations.^43^^,^^44^ Given its potential toxicity, H_2_S cellular levels must be tightly regulated. Although the (patho)physiological importance of H_2_S is well documented in eukaryotes,^2^^,^^3^^,^^45^ the issue has not been thoroughly investigated in bacterial systems.^46^

This study presents a comprehensive investigation of the H_2_S catabolic enzyme *Pa*PDO from *Pa*, one of the most significant multidrug-resistant pathogens. Our results showed the involvement of *Pa*PDO in the regulation of H_2_S levels in *Pa* and provide novel structural and functional details of the enzyme. Firstly, we demonstrated that *Pa*PDO contributes to H_2_S catabolism in *Pa*, since the Δ*pdo* deletion mutant produced ca. 4-fold higher H_2_S levels than those in PAO1, and H_2_S control levels could be restored in the Δ*pdo*(pUCP-*pdo*) complemented strain. Interestingly, *pdo* deletion did not affect *Pa* growth kinetics compared with the wild type (Figure S2), suggesting that *Pa* can tolerate, to some extent, increased endogenous sulfide levels, a feature that could be relevant for its pathogenicity.^6^^,^^9^^,^^47^ Exogenous sulfide (0.2 mM NaHS) caused only a minor growth lag phase in both the wild-type and Δ*pdo* mutant strains. On the contrary, in the presence of the same amount of sulfide, a *Staphylococcus aureus* strain deficient in the PDO-rhodanese fusion protein (CstB) exhibited a longer growth lag compared with wild-type, indicating a role of *S. aureus* CstB in counteracting the stress imposed by exogenous sulfide.^25^

**Figure 8:**
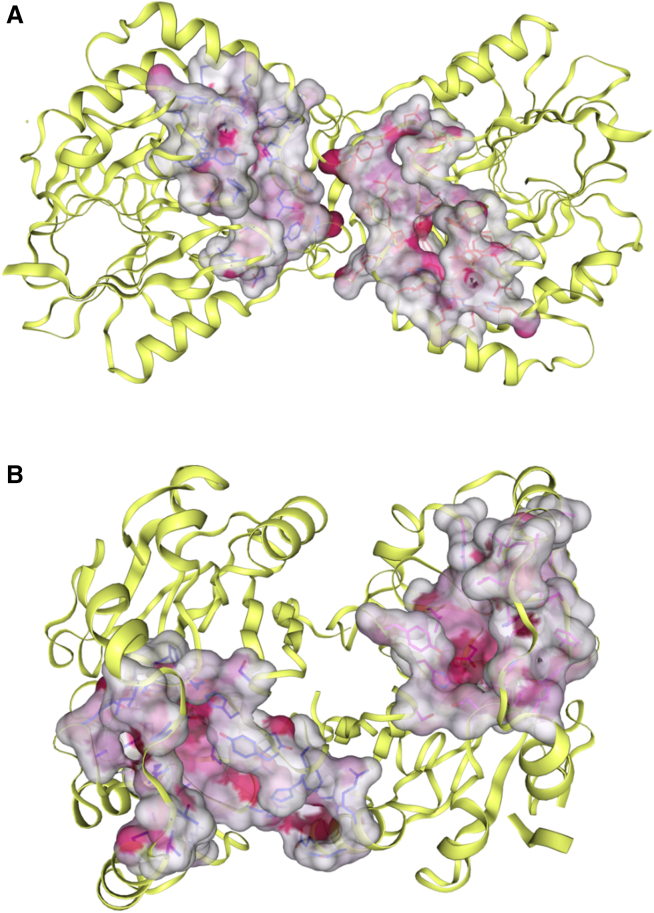
Active site cavities (A and B) Ribbon representation of the *Pa*PDO (A) and hPDO (B) dimers, with cavities containing the active site, as calculated by CB-Dock2.^48^

Our structural analysis confirmed that *Pa*PDO has the typical fold of MBL, with two β-sheets interfaced and surrounded by α-helices, forming paired halves (Figure 3A). The catalytic metal site crowns these β-sheets, with loops and α-helices providing residues for metal coordination (Figures 3B and 3C). While the overall structure and metal coordination of *Pa*PDO resemble those of hPDO (Figure S5), crucial structural differences between these two proteins could be observed. These differences support the classification of hPDO as type I PDO and of proteobacterial PDOs as type II PDO.^21^^,^^22^ In particular, both *Pa*PDO and hPDO exhibited a dimeric organization (Figure 4), but the dimerization interface in the bacterial protein was found to be larger, indicating a greater propensity for this conformation in solution (Figure 2C),^18^^,^^22^ This suggests quaternary structure as a distinguishing feature between type I and type II PDOs. Indeed, these differences can be traced to specific areas within the enzyme sequence that are absent in type I PDOs, especially in the C-terminal region, and the insertion in the loop connecting βV to βVI (R4 in *Pa*PDO, Figures 4E and S5). Is the different dimerization of PDOs linked to specific sub-functionalizations of type I and type II classes? Our analysis indicates that, while the metal-binding sites in both types of PDO are similar, their substrate accommodation differs. The cavity analysis performed with CB-Dock2^48^ highlighted that in hPDO dimers, the active sites are separated, whereas in bacterial proteins, they are contiguous (Figure 8). This suggests independent functioning of active sites in type I PDOs and a contiguous substrate-binding region in type II PDOs, potentially allowing action on larger substrates, and, thus, suggesting possible different substrate specificities. The issue requires further examination in the future.

The functional characterization herein performed revealed that *Pa*PDO can promptly catalyze the conversion of GSSH to GSH and SO_3_^2−^, using O_2_ as a co-substrate. The enzyme metabolizes GSSH with moderate affinity (*K*_m_ value > 400 μM) but high efficiency (*k*_cat_ = 165 s^−1^). While the *K*_m_ value for GSSH is consistent with those previously reported for *Pa*PDO^21^ and PDOs from other species including the hPDO, the *k*_cat_ value herein determined is higher than those previously reported.^16^^,^^18^^,^^21^^,^^25^^,^^35^ This difference may be due to differences in the experimental conditions or methodologies used. Notably, *Pa*PDO was found to display a high affinity for O_2_ (apparent *K*_*m*_ ≤ 7.5 μM (Figure S7). An excess of GSH promptly increased *Pa*PDO activity by about 2-fold, consistent with previous reports^18^^,^^35^ (Figure 5B). For the PDO from *Acidithiobacillus caldus* (*Ac*PDO), Rühl and colleagues attributed this effect of GSH to glutathionylation of some cysteine residues.^35^ Consistently, Kabil et al.^16^ speculated that GSH may react with hPDO at the level of a surface-exposed cysteine, giving rise to glutathionylated PDO. The possibility that GSH stimulates *Pa*PDO activity by acting as a positive heterotropic effector demands further investigation in the future.

The evidence that the crosstalk between gasotransmitters can affect pathogen infection^49^^,^^50^ and that the hPDO can bind to NO^51^ prompted us to investigate the interaction of *Pa*PDO with NO. The results show that this protein, (1) similar to ferrous hPDO,^51^ after full reduction of iron by GSSH, is able to bind stoichiometric NO (Figure 6A); (2) is potently, yet reversibly inhibited by NO (Figure 6C); and (3) displays low NO-dioxygenase activity (2.4 ± 0.6 s^−1^ at 2.2 μM NO) but lacks NO-reductase activity (Figure 7). It is unlikely that *Pa*PDO significantly contributes to NO detoxification in *Pa*, as the enzyme has low NO-metabolizing activity compared with other proteins involved in NO degradation in this pathogen, such as flavohemoglobin, which is endowed with high NO dioxygenase activity under aerobic conditions,^52^ or some NO reductases, which promptly detoxify NO under microaerobic conditions.^53^^,^^54^ Yet, the finding that *Pa*PDO is inhibited by NO suggests a possible interplay between the NO and H_2_S signaling pathways. In mammalian physiology, there is growing evidence for a crosstalk between NO and H_2_S (reviewed in previous studies^3^^,^^55^); depending on the experimental conditions and/or cell types, these two signaling molecules can mutually modulate their bioavailability by either inhibiting or stimulating their biosynthesis. For example, NO can inhibit the H_2_S-synthesizing enzymes CBS and CSE by heme binding^56^^,^^57^^,^^58^ or cysteine S-nitrosation,^59^ respectively, while H_2_S can inhibit endothelial nitric oxide synthase (NOS)^60^; on the contrary, NO can increase CSE and CBS expression in vascular smooth muscle cells.^61^ Similarly, sulfide has been shown to enhance NO production by increasing IL-1β-induced inducible NOS expression.^62^ Studies on the H_2_S and NO crosstalk in bacteria are still in the initial phase. Our data suggest that NO increases H_2_S levels in *Pa* by inhibiting *Pa*PDO, particularly in low-oxygen environments where NO-mediated inhibition of the enzyme was found to be more effective (Figure S9). Interestingly, in *Pa*, both H_2_S and NO play a role in biofilm formation, a key factor in its pathogenicity,^9^^,^^63^ and H_2_S synthesis is necessary for the production of virulence factors such as pyocyanin and rhamnolipids.^6^^,^^9^^,^^64^ In this context, it is tempting to speculate that the possible increase in H_2_S levels upon *Pa*PDO inhibition by NO could stimulate *Pa* virulence during the infection process when the bacterium is exposed to high NO levels produced by the host immune cells.^64^^,^^65^ Moreover, *Pa* possesses the cyanide-insensitive oxidase, a H_2_S-insensitive terminal oxidase that is more resistant to NO-induced damage than the other respiratory oxidases of this bacterium,^47^ possibly protecting *Pa* from H_2_S- and NO-derived toxicity, particularly under conditions in which high NO levels may reinforce H_2_S production.

Overall, this study reveals novel structural and functional properties of the H_2_S catabolic enzyme PDO in *Pa* and its possible role in mediating the H_2_S–NO crosstalk, positioning this enzyme as a new player in *Pa* physiology.

### Limitations of the study

The PAO1 laboratory reference strain and its Δ*pdo* mutant strain were used to demonstrate the involvement of *Pa*PDO in the modulation of sulfide levels. Our investigation represents an initial step toward a broader understanding of the role of H_2_S-oxidizing enzymes in *Pa*. Future work should extend to *Pa* clinical isolates to elucidate the physiological functions of *Pa*PDO under different conditions relevant to both chronic and acute infections and assess the impact of NO exposure on bacterial H_2_S levels.

## Resource availability

### Lead contact

Requests for further information and resources should be directed to and will be fulfilled by the lead contact, Adele Di Matteo (adele.dimatteo@cnr.it).

### Materials availability

Plasmids and strains generated in this study are available from the lead contact upon request.

### Data and code availability

• All data reported in this paper will be shared by the lead contact upon request.
• The X-ray crystallography structure of *Pa*PDO has been deposited in the PDB under accession number PDB: 9G8T.
• Any additional information required to reanalyze the data reported in this paper is available from the lead contact upon request.

## Acknowledgments

This research was supported by the Italian Ministry of University and Research with funds provided by the European Union within Next Generation EU-MUR PNRR initiatives (PRIN 2022 grant 20224BYR59 to E.F., A.G., and G.R.; the Extended Partnership Initiative on Emerging Infectious Diseases - INF-ACT, project no. PE00000007, to A.G.; and the Research project “Potentiating the Italian Capacity for Structural Biology Services in Instruct-ERIC” - ITACA.SB, project no. IR0000009), by Fundação para a Ciência e a Tecnologia I. P., Portugal, through iNOVA4Health (UIDB/04462/2020, UIDP/04462/2020) and LS4FUTURE Associated Laboratory (LA/P/0087/2020) to J.B.V., and by Sapienza University of Rome, Italy (grants RM122181698FC992 and RP124191037022F7) to E.F. J.B.V. received support from Horizon 2020, European Union (MSCA-RISE-2018): ProMeTeus (ID: 823780). We acknowledge Elettra Sincrotrone Trieste for providing access to its synchrotron radiation facilities, and we thank Nicola Demitri for assistance in using beamline XRD2.

## Author contributions

Conceptualization, A.D.M., A.G., E.F., G.R., G.G., and J.B.V.; methodology, A.D.M., A.G., E.F., G.R., and G.G.; investigation, F.G., F.T., M.R.N., L.C., and M.M.; writing – original draft, F.G., F.T., G.R., G.G., A.D.M., and E.F.; writing – review & editing, F.G., F.T., M.R.N., L.C., M.M., C.T.A., G.G., J.B.V., G.R., A.D.M., E.F., and A.G.; funding acquisition, A.G., G.R., J.B.V., and E.F.; resources, A.D.M., E.F., and A.G.; supervision, A.D.M., A.G., E.F., G.R., G.G., and J.B.V. All authors have read and agreed to the published version of the article.

## Declaration of interests

The authors declare no competing interests.

## STAR★Methods

### Key resources table

**Table:** 

REAGENT or RESOURCE	SOURCE	IDENTIFIER
**Bacterial and virus strains**		

*Escherichia coli* DH5a	Grant et al.^66^	N/A
*Escherichia coli* S17.1 λ*pir*	Simon et al.^67^	N/A
*Pseudomonas aeruginosa* PAO1	ATCC	ATCC15692
*Pseudomonas aeruginosa* PAO1 Δ*pdo*	This study	N/A

**Chemicals, peptides, and recombinant proteins**		

Oxidized L-glutathione disodium salt	MERCK	G4626; CAS: 103239-24-3
Reduced L-glutathione	MERCK	G6013; CAS: 70-18-8
Sodium sulfide nonahydrate	MERCK	208043; CAS: 1313-84-4
Ascorbate oxidase from *Cucurbita* sp.	MERCK	A0157; CAS: 9029-44-1
Sodium L-ascorbate	MERCK	11140; CAS: 134-03-2
5,5’-dithiobis-2-nitrobenzoic acid	MERCK	D8130; CAS: 69-78-3
3-(2-pyridyl)-5,6-bis(4-phenylsulfonic acid)-l,2,4-triazine	MERCK	160601; CAS: 63451-29-6
Desossiribonucleasi I	MERCK	DN25, CAS: 9003-98-9
Ribonucleasi A	MERCK	R5250; CAS: 9001-99-4
Lysozyme	MERCK	L6876; CAS: 12650-88-3
Isopropyl-β-D-thiogalactoside	Enzo Life Sciences	ALX-582-002; CAS: 367-93-1
Kanamycin monosulfate	MERCK	BP861; CAS: 25389-94-0
Ferrozine (3-(2-Pyridyl)-5,6-diphenyl-1,2,4-triazine-p,p′-disulfonic acid monosodium salt hydrate)	MERCK	160601; CAS: 63451-29-6
Ampicillin (Ap)	MERCK	A9518 – CAS 69-52-3
L-cysteine	MERCK	C1276 – CAS 52-89-1
NaHS	MERCK	161527 – CAS 207683-19-0
Nalidixic acid	MERCK	N4382 – CAS 3374-05-8
Chloramphenicol	MERCK	C0378 – CAS 56-75-7
Carbenicillin	MERCK	C1389 – CAS 4800-94-6
Sucrose	MERCK	S0389 – CAS 57-50-1
FastDigest Green Buffer (10X)	Thermo Scientific™	B72
FastDigest XhoI	Thermo Scientific™	FD0694
FastDigest EcoRI	Thermo Scientific™	FD0274
FastDigest XbaI	Thermo Scientific™	FD0685
*Pa*PDO synthetic gene	GenScript	N/A

**Critical commercial assays**		

Pierce™ BCA Protein Assay Kits	Thermo Scientific	23226
Wizard® SV Gel and PCR Clean UP system	Promega	A9282
Wizard® Plus SV Minipreps DNA Purification System	Promega	A1460
T4 DNA ligase (Ligase and Buffer)	Promega	M1801
Hydrogen Sulfide Test Strips	Fluka	06728-25STRIPS-F
GoTaq® G2 Flexi DNA Polymerase	Promega	M7805

**Deposited data**		

PDO structure	This paper	PDB: 9G8T

**Oligonucleotides**		

FW*pdo*UP 5’-CCGCTCGAGGTTGCTGCGACGCCATCC-3’	This paper	N/A
RV*pdo*UP 5’-GGAATTCTTTCAACATGGAGGTTCCTTG-3’	This paper	N/A
FW*pdo*DW 5’-GGAATTCCCGCCGGTGGAAGGCAAC-3’	This paper	N/A
RV*pdo*DW 5’-GCTCTAGAGGCGACCACCGCGCCG-3’	This paper	N/A
*pdo*_FW 5’-GGAATTCATGTTGAAACCCGACATCACT-3’	This paper	N/A
*pdo*_RV 5’-GCTCTAGATCAGAACAGATCCAGCGG-3’	This paper	N/A
FW*pdo*INT 5’-ATCTTCCTGCAGCGCGAAC-3’	This paper	N/A
RV*pdo*INT 5’-TCGCGGACGTGCACGTTG-3’	This paper	N/A
RVM13 5’-CAGGAAACAGCTATGAC-3’	This paper	N/A

**Recombinant DNA**		

pUCP18 plasmid	Schweizer^68^	GenBank: U07164.1
pUCP-*pdo* plasmid	This paper	N/A
pDM4 plasmid	Milton et al.^69^	N/A
pDM4Δ*pdo*	This paper	N/A

**Software and algorithms**		

GraphPad Prism 9.0	N/A	https://www.graphpad.com/updates/prism-900-release-notes
XDS	Kabsch^70^	https://xds.mr.mpg.de/
AIMLESS	Evans et al.^71^	https://www.ccp4.ac.uk/
PHASER	McCoy et al.^72^; Liebschner et al.^73^	https://phenix-online.org/
Phenix_refine	Afonine et al.^74^; Liebschner et al.^73^	https://phenix-online.org/
COOT	Emsley et al.^75^	https://www.ccp4.ac.uk/
Chimera	Pettersen et al.^76^	https://www.cgl.ucsf.edu/chimera/
CB-DOCK2	Liu et al.^48^	https://cadd.labshare.cn/cb-dock2/index.php

### Experimental model and study participant details

Bacterial strains and plasmids used in this study are listed in the key resources table. *E. coli* and *Pa* strains were routinely grown at 37°C in Luria Bertani medium (LB), in static or shaking cultures (200 rpm) or in LB supplemented with 15 g/L agar. When required, media were supplemented with 200 μM L-cysteine or 200 μM NaHS, and antibiotics were added at the following concentrations: ampicillin (Ap) 100 μg/mL (E. coli); nalidixic acid (Nal) 15 μg/mL (*E. coli*); chloramphenicol (Chl) 30 μg/mL (E. coli) or 375 μg/mL (*Pa*); carbenicillin (Cb) 300 μg/mL (*Pa*).

### Method details

#### Preparation of NO solutions

NO solutions were prepared by equilibrating in a tonometer pure NO gas (1 atm at 20°C) with 20 mL ultra-pure water previously degassed by N_2_ bubbling for 30 min. NO concentration in solution was determined by spectrophotometric titration of fully reduced beef heart cytochrome *c* oxidase, which binds NO with a 1:1 stoichiometry,^77^ yielding a characteristic absorption spectrum.^78^

#### Preparation of glutathione persulfide (GSSH) solutions

Stock solutions of H_2_S were prepared by dissolving 50-60 mg of Na_2_S crystals in degassed 200 mM Tris-HCl pH 8.0 under N_2_ atmosphere as reported in.^79^ Sulfide concentration was determined using the Ellman’s reagent (5,5’-dithiobis-2-nitrobenzoic acid, DTNB.^80^ Glutathione persulfide (GSSH) was prepared by incubating at room temperature for 1 h under anaerobic conditions a solution of H_2_S mixed with a 5-fold excess of oxidized glutathione (GSSG) in 200 mM Tris-HCl pH 8.0. GSSG reacts with H_2_S according to the following equation (Equation 2):

(Equation 2) $$\text{GSSG}+H_{2}S\;\to \;\text{GSSH}+\text{GSH}$$

GSSH concentration was determined by performing the cold cyanolysis assay^81^ in a Cary 60 UV-VIS spectrophotometer (Agilent Technologies Inc, Santa Clara, California, USA).

#### Recombinant DNA techniques

Plasmid DNA preparation, purification of DNA fragments, restriction, ligation and transformation in *E. coli* DH5α^66^ or *E. coli* S17.1λ*pir*^67^ competent cells were performed following standard procedures.^82^ DNA amplification was performed by PCR using the GoTaq Polymerase (Promega). FastDigest restriction enzymes were purchased from Thermo Fisher Scientific. Ligation of DNA fragments was performed using T4 DNA Ligase (Promega). Plasmids were introduced into *Pa* by transformation or bi-parental conjugation using *E. coli* S17.1λ*pir* as the donor strain.^82^ All plasmids generated in this study were verified by restriction analysis and DNA sequencing.

#### Plasmids construction

The pDM4Δ*pdo* plasmid for the deletion of the *pdo* gene in *Pa* PAO1 (ATCC15692) was generated as follows: ca. 550 base pairs (bp) upstream and downstream regions of the *pdo* gene were PCR amplified from the *Pa* PAO1 genome by using the primer pairs FW*pdo*UP (CCGCTCGAGGTTGCTGCGACGCCATCC) and RV*pdo*UP (GGAATTCTTTCAACATGGAGGTTCCTTG), for the upstream region, and FW*pdo*DW (GGAATTCCCGCCGGTGGAAGGCAAC) and RV*pdo*DW (GCTCTAGAGGCGACCACCGCGCCG) for the downstream region (restriction sites in the oligonucleotide sequences are underlined). The resulting amplicons (*i.e.* upstream and downstream regions of *pdo*) were cloned together in pBluescript II KS(+) (Stratagene) by using the restriction enzymes XhoI/EcoRI, for the upstream region, and EcoRI/XbaI, for the downstream region. The joined upstream and downstream regions were subcloned to pDM4^69^ by using XhoI/XbaI restriction enzymes.

The pUCP-*pdo* plasmid for constitutive expression of *pdo* was generated as follows: the *pdo* gene was PCR amplified from *Pa* PAO1 genome by using the primer pair *pdo*_FW (GGAATTCATGTTGAAACCCGACATCACT) and *pdo*_RV (GCTCTAGATCAGAACAGATCCAGCGG). The resulting amplicon was cloned into the pUCP18 plasmid^68^ by using the EcoRI-XbaI restriction enzymes and verified by sequencing (Figure S1).

#### Generation of the *Pa* Δ*pdo* mutant

The *Pa* mutant strain deleted in the *pdo* gene (Δ*pdo*) was generated by allelic exchange using the pDM4-derivative plasmid pDM4Δ*pdo*, as previously described.^83^ The pDM4Δ*pdo* plasmid was transferred from the *E. coli* S17.1λ*pir* donor strain to *Pa* PAO1 by conjugation.^82^ Clones carrying the pDM4Δ*pdo* chromosomal insertion were selected on LB agar plates supplemented with 375 μg/mL Cm and 15 μg/mL Nal. Plasmid excision from the chromosome was then selected on LB agar plates supplemented with 10% (w/v) sucrose. Deletion of the *pdo* gene was verified by PCR analysis (Figure S1).

#### H_2_S quantification in bacterial cultures

H_2_S in cultures of PAO1 wild type and its isogenic Δ*pdo* mutant, carrying or not the pUCP18 or pUCP-*pdo* plasmids, was quantified by using the lead acetate detection method.^34^ Overnight cultures were diluted 1:100 in LB, and 100 μL aliquots were dispensed in 96-well microtiter plates. Paper strips saturated with 2% Pb(Ac)_2_ were affixed over the wells, in the gas phase above the liquid cultures. An adhesive plastic sheet (AriaMx Adhesive Plate Seals, Agilent) impermeable to H_2_S^12^ was used to seal the wells and prevent H_2_S leakage. The reaction between H_2_S released from the culture and lead acetate results in a brown stain on the paper strip, whose intensity is proportional to the level of H_2_S produced. After 24 h incubation at 37°C in static conditions, paper strips were gently removed, scanned, and measured *via* densitometric analysis by using the ImageJ software. Relative H_2_S production was determined as the densitometric value obtained for each culture corrected for the background value (*i.e.* the densitometric value obtained for the non-inoculated medium).

#### Protein expression and purification

The synthetic gene encoding PDO from *Pa* (*Pa*PDO, Uniprot: PA2915) was purchased from GenScript (Piscataway, NJ, USA) and cloned into the pET-30a expression vector with the 6xHis at the N-terminus*.* The protein was expressed in *E. coli* BL21 (DE3). Bacteria were grown in LB or M9 minimal medium supplemented with 100 μM Fe(NH_4_SO_4_)_2_ and 30 μg/mL of kanamycin at 37°C to A_600_ = 0.6 OD. The expression of PDO was induced by addition of 0.2 mM isopropyl-β-D-thiogalactoside (IPTG), and bacteria were grown overnight at 22°C. The bacterial pellet obtained after low-speed centrifugation was resuspended in 50 mM Tris-HCl pH 8.0, 200 mM NaCl, 20 mM imidazole containing 1 mM PMSF and lysozyme, DNase I, RNase A, and then sonicated on ice (3 s on and 7 s off). The supernatant was collected by centrifugation at 17400 x g for 40 min at 4°C and loaded on a HisTrap FF (GE Healthcare, Chicago, IL, USA) column, pre-equilibrated with 50 mM Tris-HCl pH 8.0, 200 mM NaCl, 50 mM imidazole. Proteins were eluted with an imidazole gradient (50 - 500 mM) and collected fractions were buffer exchanged with 20 mM Tris-HCl pH 8.0, 200 mM NaCl with a HiTrap Desalting column (GE Healthcare, Chicago, Illinois, USA). Protein concentration was determined with the BCA assay (Thermo Scientic). The purification yield was ≈ 40 mg protein per liter of culture, in both LB and M9 media.

#### Iron quantification in *Pa*PDO

Iron content in isolated *Pa*PDO was measured using the 3-(2-pyridyl)-5,6-bis(4-phenylsulfonic acid)-l,2,4-triazine (ferrozine) assay.^84^ Briefly, *Pa*PDO was denatured by incubation with 37% HCl at 80°C for 30 min and then centrifuged for 5 min at 16200 × g. To determine the total iron content, the supernatant was mixed with an ammonium acetate-oversaturated solution containing 10 mM ferrozine and 75 mM ascorbate, which reduces the iron, and then incubated at room temperature for 20 min in the dark. To determine the ferrous iron content in the “as prepared” and GSSH-treated *Pa*PDO used for NO binding experiments, the samples were mixed with an ammonium acetate-oversaturated solution containing 10 mM ferrozine but water in place of ascorbate, followed by incubation at room temperature for 20 min in the dark. The amount of the Fe II-ferrozine complex was then measured spectroscopically by recording the absorbance at 562 nm. The iron concentration was determined using a standard curve obtained with Fe(NH_4_SO_4_)_2_ (Figure S3A). The *Pa*PDO purified from LB cultures had an estimated iron occupancy of about 50%, while the protein obtained from M9 cultures supplemented with 100 μM Fe(NH_4_SO_4_)_2_ of about 70%. Fractional Fe occupancy was taken into account when catalytic activity was determined. The ferrous iron content in the “as isolated” *Pa*PDO varied from preparation to preparation up to about 50%, whereas in the GSSH-treated *Pa*PDO it matched the total iron content (Figure S3B).

#### Size-exclusion chromatography

Size-exclusion chromatographic (SEC) analysis was performed using a Superdex 75 10/300 column (GE-Healthcare, Chicago, Illinois, USA) in 20 mM Tris-HCl pH 8.0, 200 mM NaCl. The column was connected to an HLPC system (KNAUER, Berlin, Germany), and the flow rate was fixed at 1 mL/min. The column was calibrated using the following protein standards (Cytiva, Marlborough, MA, USA): albumin (66.5 kDa), ovalbumin (43.0 kDa), chymotrypsinogen (25.0 kDa), ribonuclease-A (13.7 kDa), which eluted at 10.2, 11.1, 12.9 and 13.8 mL, respectively.

#### Far-UV CD analysis

Far-UV circular dichroism (CD) measurements were carried out using a Jasco J710 instrument (Jasco Inc., Easton, MD, USA) equipped with a Peltier temperature controller. CD spectra of *Pa*PDO (0.2 mg/ml) were collected at 20°C in the far-UV region (200-250 nm) in 20 mM Tris-HCl pH 8.0, 200 mM NaCl buffer in a 1-mm quartz cell (scanning speed of 100 nm/min, average of three acquisitions). Thermal denaturation was followed by monitoring the CD signal at 210 nm (1°C/min thermal ramp, from 20°C to 90°C). GraphPad Prism 9.0 was used for graphing and data analysis.

#### Crystallization and X-ray data collection

*Pa*PDO crystallization conditions were initially screened automatically with the Oryx-4 crystallization robot (Douglas Instruments). Single crystals were observed in condition No. 9 of the Morpheus crystallization screen (Molecular Dimensions) and were further reproduced and optimized manually by the hanging drop vapor diffusion method.

Best diffracting crystals grew in 1 day and were obtained by mixing 1.5 μL of protein solution (8 mg/mL) with 1 μL of the reservoir solution containing 0.06 M Divalents (0.03 M magnesium chloride hexahydrate; 0.03 M calcium chloride dihydrate), 0.1 M Buffer System 3 pH 8.5 (0.05 M Tris (base); 0.05 M BICINE), 30% v/v Precipitant Mix 1 (20% v/v PEG 500 MME; 10% w/v PEG 20000), and equilibrating the obtained solution versus 500 μL of reservoir solution at 20°C.

Flash-frozen crystals were exposed to X-rays at the XRD2 Beamline of ELETTRA Synchrotron (Trieste, Italy). Diffraction data were collected at 1.000 Å wavelength with an oscillation range of 0.5°. Data were processed and scaled with XDS^70^ and AIMLESS.^71^ The best crystal diffracted at 2.06 Å resolution and belonged to the P3_1_2_1_ space group with one molecule *per* asymmetric unit and 45% of solvent. Full statistics are reported in Table S1.

#### Structure solution and refinement

*Pa*PDO structure was solved by molecular replacement with Phaser^72^ as implemented in Phenix^73^ using the structure of PDO from *P. putida* (PDB code: 4YSK,^22^ as the search model. Refinement and model building were performed with Phenix_refine^74^ and COOT.^75^ The protein crystallized as a dimer, with the 2-fold axis of the dimer corresponding to the crystallographic 2-fold symmetry. Therefore, the model consists of 1 molecule in the asymmetric unit and the homodimer (biological assembly) is generated by applying crystallographic symmetry. X-ray fluorescence spectroscopy (Figure S4) and anomalous signal analysis were used to assign the nature of the metal in the metal-binding site. Although a mixed situation was observed, compatible with the presence of Zn, Ca and Ni, but with a prevalence of the first metal, it was decided to model 1 Zn atom with 100% occupancy. Thus, the final model comprises one protein molecule consisting of 287 residues, 1 Zn atom and 78 water residues. Residues 208-215 could not be fitted in the electron density map. Refinement and model building statistics are reported in Table S1. Coordinates and structure factors have been deposited in the Protein Data Bank with accession code 9G8T. Figures were prepared with Chimera.^76^ Cavities were detected by CB-DOCK2.^48^

#### Persulfide dioxygenase activity and NO interaction experiments

The persulfide dioxygenase activity of *Pa*PDO was assessed by oxygraphic measurements, using a high-resolution respirometer (Oxygraph-2k, Oroboros Instruments GmbH, Innsbruck, Austria) equipped with two 1.5-mL chambers. Assays were carried out at 25°C in 100 mM sodium phosphate pH 7.4 in the absence of light due to photosensitivity of GSSH. The reaction was initiated by adding 5-7 nM *Pa*PDO holoenzyme to an air-equilibrated solution containing GSSH at varied concentrations. To determine the *K*_m_ for O_2_, measurements were carried out in the presence of 160 μM GSSH at varied O_2_ concentrations. To perform simultaneous measurements of O_2_ and NO in solutions, a NO-selective amperometric sensor ISO-NO (World Precision Instruments, Sarasota, Florida, USA) was connected to the oxygraph. Amperometric traces were recorded using the software DatLab 6.0 (Oroboros Instrument) and LabScribe2 (World Precision Instruments). The effects of NO and GSH on *Pa*PDO activity were measured in the presence of 160 μM GSSH. In the assays with NO, NO was added when the O_2_ concentration was 200 μM.

The NO:PDO binding measurements were performed under anaerobic conditions achieved by thorough nitrogen flushing and addition of 1 U/mL ascorbic oxidase and 5 mM ascorbate.^85^^,^^86^ Briefly, 35 μM *Pa*PDO holoenzyme was previously degassed under N_2_ flux for 10 minutes and then incubated for 5 minutes with a 10-fold excess of GSSH (350 μM) at room temperature. Afterwards, the treated protein was added to degassed Na phosphate 100 mM pH 7.4 containing 4.4 μM NO and 160 μM GSSH, and the concentration of NO in solution was monitored by using the NO selective electrode. The iron *Pa*PDO stoichiometry was calculated by subtracting from the amount of NO, disappearing from solution upon addition of the GSSH-reduced enzyme, the amount of NO consumed by the same amount of GSSH alone, as independently assessed in a control assay (Figure S8). *Pa*PDO-NO dioxygenase activity was calculated using a rise and fall equation with baseline time course for GraphPad Prism 9.0, as reported in.^87^

### Quantification and statistical analysis

Except for crystallographic data refinement, statistical analyses were performed with the software GraphPad Prism 9.0 (www.graphpad.com). The unpaired *t*-test (single-comparison) was used, and data sets with *P* values lower than 0.05 were considered statistically significant.
