# Supplementary material for: Structure and function of persulfide dioxygenase from Pseudomonas aeruginosa: Implications on H2S homeostasis and interplay with nitric oxide
Source: iScience. 2025 Dec 30;29(2):114586. doi: 10.1016/j.isci.2025.114586 (PMC12856154; doi:10.1016/j.isci.2025.114586)
Supplement: Document S1. Figures S1–S9 and Table S1 [file mmc1.pdf]

iScience, Volume *29*

Supplemental information

Structure and function of persulfide dioxygenase from *Pseudomonas aeruginosa*: Implications on H_2S homeostasis and interplay with nitric oxide

Francesca Giordano, Francesca Troilo, Martina Roberta Nastasi, Lorenzo Caruso, Marta Mellini, Carlo Travaglini-Allocatelli, Giorgio Giardina, João B. Vicente, Giordano Rampioni, Adele Di Matteo, Elena Forte, and Alessandro Giuffrè

Supplementary material

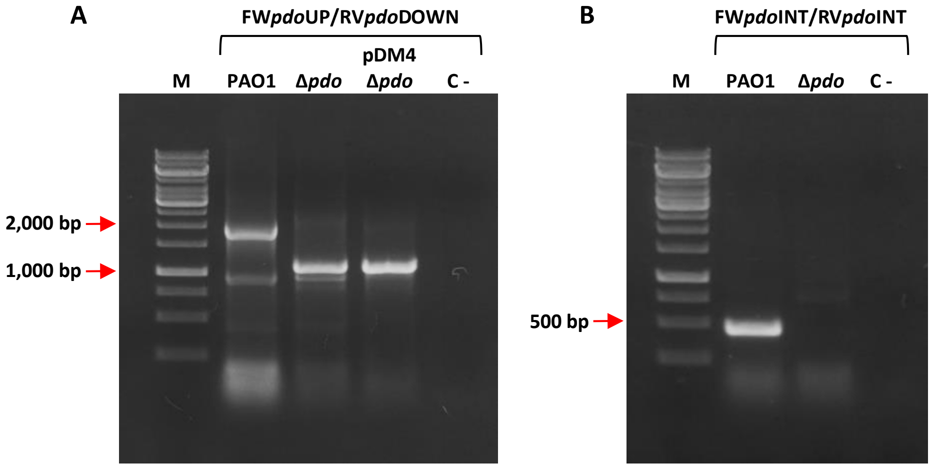

C

pUCP-*pdo* sequenced region

TGATTACGAATTC***ATG****TTGAAACCCGACATCACTGCCTTCTTCGACCCGGCCACCTCCACCTACAGCTACG*
TGGTGCGCGACCCGTCCAGCCGCGCCTGCGCCATCGTCGACCCGGTGCTCGACTACGACCCGGCCGCCGGG
CGCACCAGTCATGCCAGCGCCGAACGCCTGATCGCCCACGTTCGCCAGCACGACCTGCAGGTGGAATGGCT
GCTGGAGACCCACGTGCACGCCGACCACCTGTCGGCGGCCATCTTCCTGCAGCGCGAACTGGGCGGGTGCC
TGGCCATCGGCGCGCGAATCACCCAGGTGCAGGCGAAATTCTCCGGCCTGTTCAACCTCGGCGAGGCGTTC
CCGGTGGACGGCCGCCAGTTCGAGCACCTGTTCGAGGACGGCGAGTCGTTCCGCATCGGCGCCCTGGAATG
CCGCGCGCTGCATACCCCGGGGCATACCCCGGCGTGCATGACCTACCTGGTCGGCGACAGCGCCTTCGTCG
GCGATACCCTGTTCATGCCCGACTACGGCACCGCCCGCTGCGATTTCCCCGGCGGCGACGCGCGCCAGCTG
TATCGCTCGATCCAGCGCCTGTTCGCGCTACCCGACGCGACCCGCCTGTTCATGTGCCACGACTACACGGC
TCCCGGCCGCGACGAGCACCGCTGCGAAACCAGCGTCGGCGAGCAGCGCCGGCACAACGTGCACGTCCGCG
AAGGCGTCGACGAGGAGGCGTTCGTCGCCATGCGCCAGCAGCGCGATGCCACCCTCGGCATGCCGACCCTG
ATGCTGCCGGCGATCCAGGTCAACATGCGCGGCGGCAACCTGCCGCCGGTGGAAGGCAACGGGGTGCGTTA
*CCTGAAGATCCCGCTGGATCTGTTC****TGA***TCTAGAGTCGACCTGCAGGCATGCAAGCTTGGCACTGG

Figure S1. Validation of the *pdo* mutant and pdo-expressing plasmid. (A) and (B) Gel electrophoresis analysis of PCR products performed to verify the deletion of the *pdo* gene from PAO1 chromosome. (A) Amplicons obtained on the PAO1 and the Δ*pdo* chromosomes, or the pDM4Δ*pdo* plasmid, by using primers FW*pdo*UP (CCGCTCGAGGTTGCTGCGACGCCATCC) and RV*pdo*DOWN (GCTCTAGAGGCGACCACCGCGCCG). Expected products length was 1,898 bp for PAO1, and 1,080 bp for Δ*pdo* and pDM4Δ*pdo.* (B) Amplicons obtained on the PAO1 and the Δ*pdo* chromosomes by using primers FW*pdo*INT (ATCTTCCTGCAGCGCGAAC) and RV*pdo*INT (TCGCGGACGTGCACGTTG). Expected product length was 458 bp for PAO1, while no product was expected in the Δ*pdo* mutant as these primers anneal in an internal region of the *pdo* gene. M, molecular weight marker (Generuler 1 kb DNA ladder, GeneDirex); C -, no DNA template added. (C) DNA sequence obtained by Sanger sequencing of the pUCP-*pdo* plasmid using primer RVM13 (CAGGAAACAGCTATGAC), which anneals 26 bp upstream of the EcoRI restriction site in pUCP18. The obtained sequence includes the complete *pdo* gene and a portion of the pUCP18 vector. Alignment between the sequenced plasmid region and the theoretical sequence revealed complete correspondance (verified with the Clustal Omega sequence alignment tool). The pUCP18 sequence is underlined; recognition sites for the EcoRI and XbaI restriction enzymes used for *pdo* cloning into pUCP18 are shaded in green; the *pdo* gene sequence is shown in italics, with start and stop codons in bold

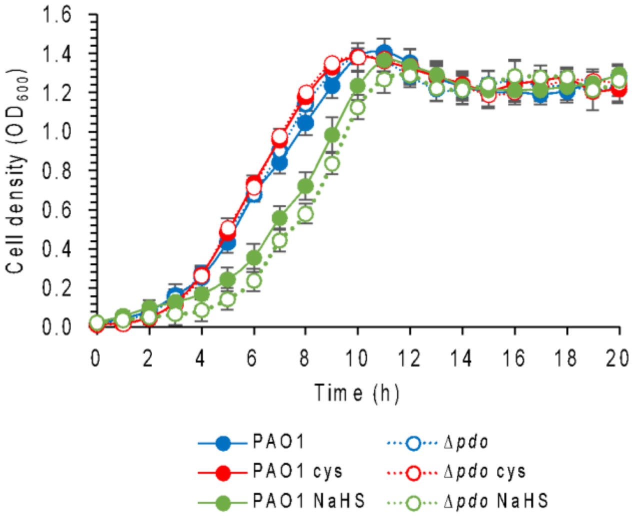

Figure S2. **Growth curves of *P. aeruginosa* PAO1 and its isogenic Δ*pdo* mutant.** Growth curves of *P. aeruginosa* PAO1 (solid lines, full circles) and its isogenic Δ*pdo* mutant (dashed lines, empty circles). Strains were grown in 96-well microtiter plates in LB (blue lines) or in LB supplemented with 200 µM L-cysteine (cys; red lines) or 200 µM NaHS (green lines). Mean and standard deviations

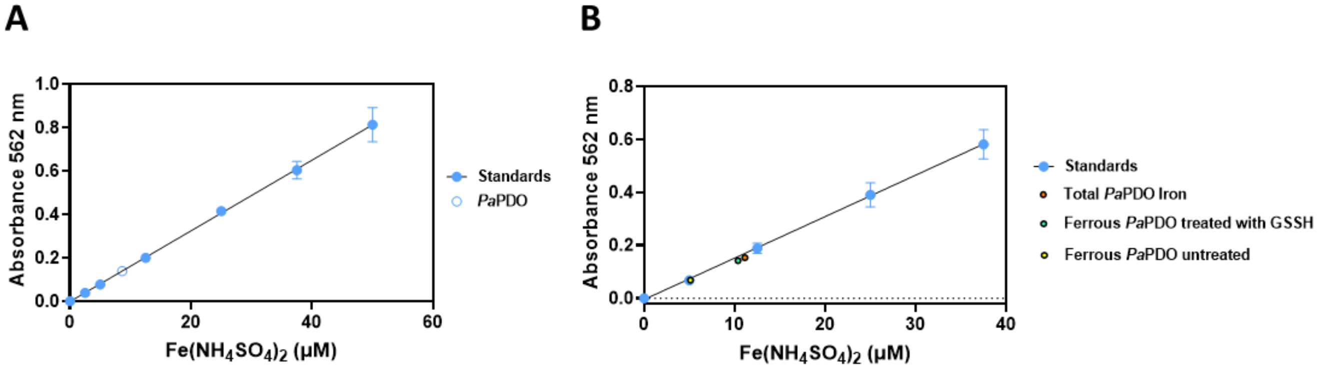

Figure S3. Ferrozine Assay for Iron quantification of *Pa*PDO. Standard curve: solid blue circles represent the absorbance at 562 nm measured for increasing concentrations of Fe^{2+} (as $Fe(NH_4SO_4)_2$) complexed with ferrozine. (A) Total iron content and iron occupancy estimation in the "as-prepared" *Pa*PDO. The empty blue circle indicates the interpolated absorbance value corresponding to the total iron content in the *Pa*PDO sample. The measured iron concentration (8.3 µM), with respect to *Pa*PDO monomer concentration (11.3 µM), indicates a stoichiometry of approximately 0.7 mol Fe per mol *Pa*PDO monomer, i.e. ~70% of the protein is in the holo form. (B) Ferrous iron content in GSSH–treated (green circle) and untreated *Pa*PDO (yellow circle). *Pa*PDO total iron content (orange circle). In the presence of GSSH, the iron is almost fully reduced (11 µM total iron, 10,3 µM ferrous iron in GSSH-treated *Pa*PDO and 5,1 µM in the untreated sample).

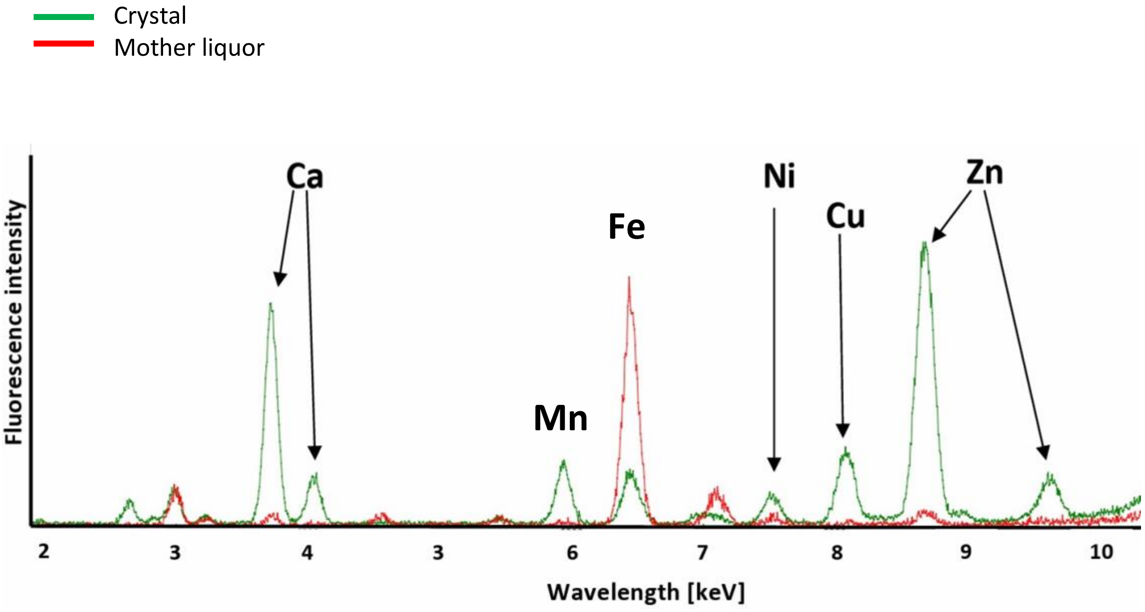

Figure S4. **X-ray fluorescence spectrum of *Pa*PDO crystals.** The emission spectrum of the crystal and the mother liquor are shown in green and red, respectively. Spectra indicates a mixed situation with a prevalence of Zn and Ca ions.

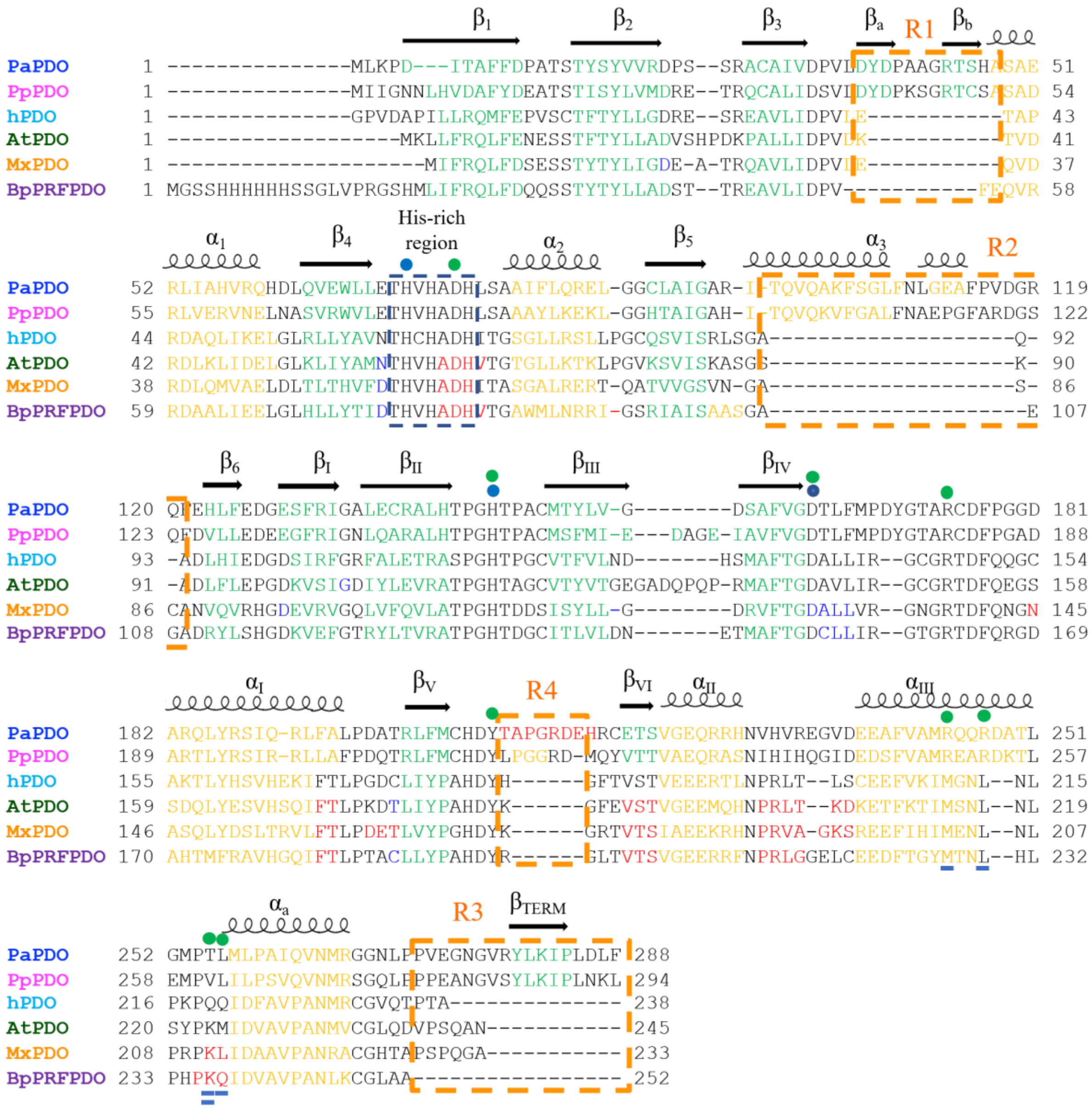

Figure S5. Sequence comparison of PDO from *P. aeruginosa*, *P. putida* and hPDO. Sequence comparison of PDO from *P. aeruginosa* (*Pa*PDO, Uniprot: Q9HZT2) P. putida (*Pp*PDO, PDB code: 4ysk, Uniprot: A5VWI3), hPDO (hPDO, PDB code: 4chl, Uniprot: O95571), M. xanthus (MxPDO, PDB code: 4ysb, Uniprot: Q1D4C9), A. thaliana (AtPDO, PDB code: 2gcu, Uniprot: Q9C8L4), B. phytofirmans (BpPRF, PDB code: 5ve3, Uniprot: B2TEQ2). The residues involved in the metal coordination are marked with blue dots; the residues involved in the interaction with GSH, according to Sattler et al. 2015 [28], are marked with green dots. Among them, underlined residues are conserved only in *Pa* and *Pp*PDO, the amino acid underlined with 2 lines is not conserved

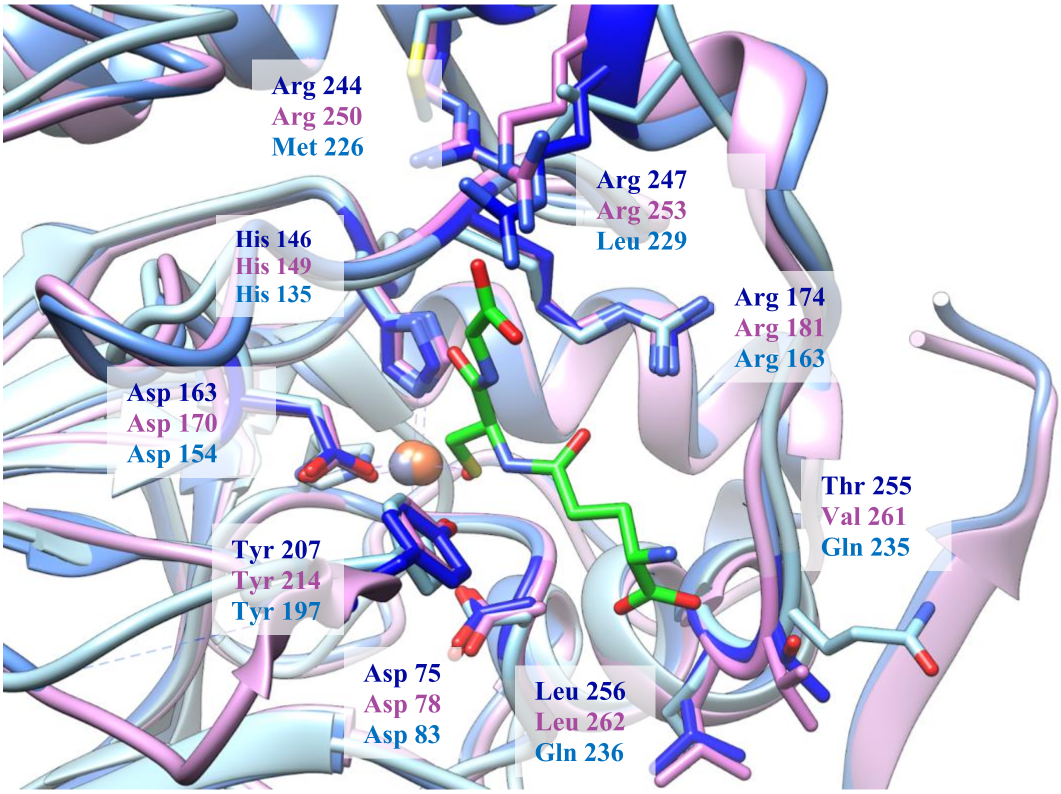

Figure S6. **Comparison of the PDO active sites.** Close view of the active sites of the superposed structures of hPDO (light blue), *Pa*PDO (cornflower blue) and *Pp*PDO (plum) with the substrate (GSH, light green) from *Pp*PDO structure (PDB code: 4ysl). The residues involved in the interaction with GSH, according to Sattler et al. 2015, are represented in light purple in *Pp*PDO, in blue in *Pa*PDO and in light blue in hPDO.

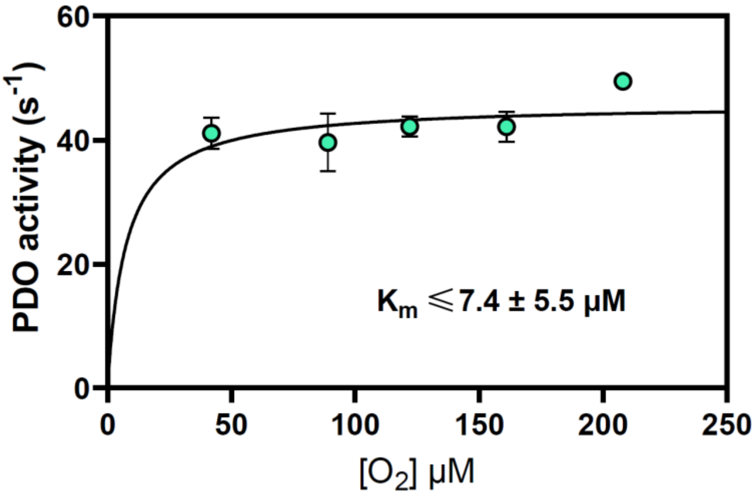

Figure S7. **Apparent Km for oxygen.** *Pa*PDO activity versus O2 concentration was determined at 25 °C in 100 mM Na phosphate pH 7.4 in the presence of 160 µM GSSH. The activity was determined at different O2 concentrations (50- 250 µM) obtained by bubbling the solution with N_2 flux for different seconds. PDO activity is about the same for all the O2 concentrations, the higher limit of the Km (O_2) was determined.

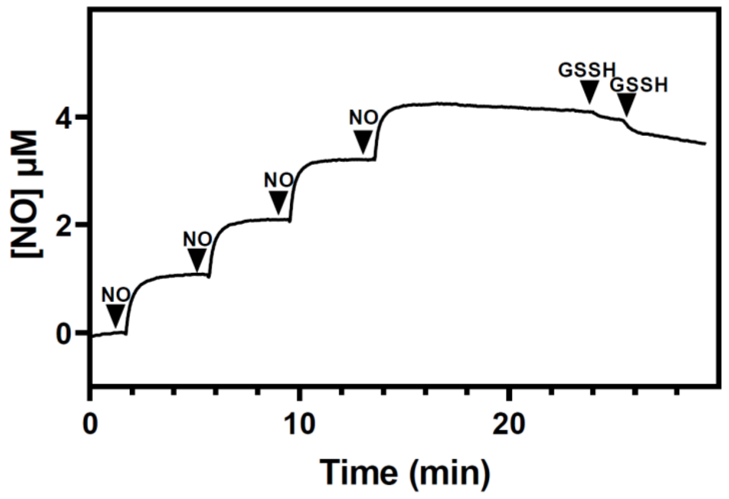

Figure S8. **Effect of GSSH on NO concentration under anaerobic conditions.** Four consecutive aliquots of 1.1 µM NO were added to degassed Na phosphate 100 mM buffer, pH 7.4, in the presence of 1 U/ml ascorbic oxidase and 5 mM ascorbate. The addition of 5 µM and 10 µM GSSH aliquots to the chamber results in a small decrease in NO concentration compared to *Pa*PDO.

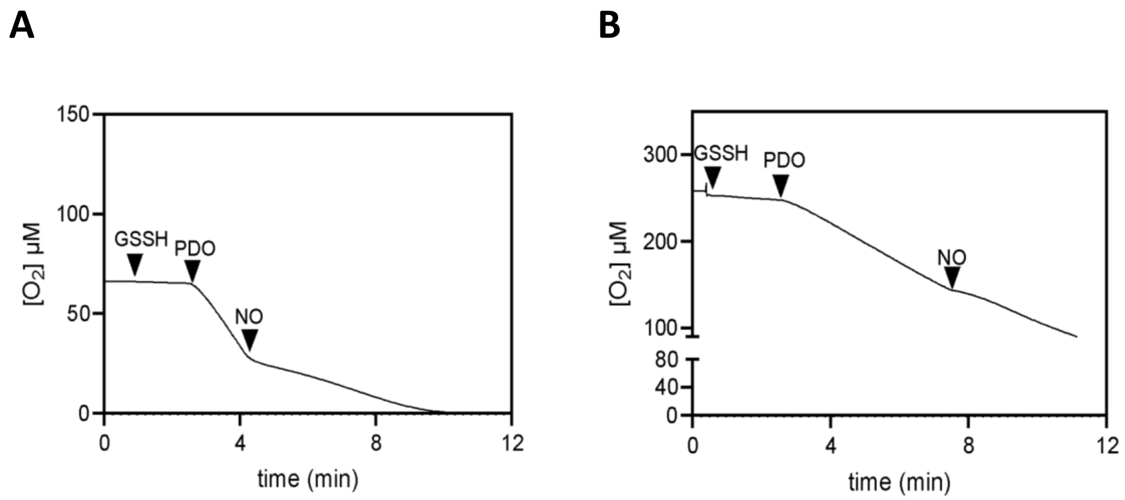

Figure S9. ***Pa*PDO inhibition by NO at low and high oxygen**. Oxygraphic traces of *Pa*PDO (5.3 nM holoprotein) in the presence GSSH (160 µM). (A) 1 µM NO was added at 20 µM oxygen. (B) 1 µM NO was added at 130 µM oxygen. *Pa*PDO was more potently inhibited by NO at low oxygen levels

Table I. X-ray data collection and refinement statistics for *Pa*PDO.

**Data collection**	
Beamline	ELETTRA - XRD2
Wavelength (Å)	1.000
Space group	P 31 2 1
Cell dimensions (Å, °)	77.24 77.24 83.30; 90 90 120
Resolution range (Å)	66.89 – 2.06 (2.14 - 2.06)
CC(1/2)(%)	99.9 (85.5)
Mean I/sigma (I)	18.2 (2.2)
Completeness (%)	100 (100)
Reflections	
Total reflections	317221 (15700)
Multiplicity	17.5 (17.7)
Wilson plot *B* value	45.7
**Refinement**	
Resolution range	66.89 – 2.06
R_{work}/R_{free} *(%)	19.2 (26.3) / 23.9 (30.2)
Average B-factor (Å^2) (no. of atoms)	
Overall	61.93 (2285)
Protein	62.01 (2197)
Waters	57.02 (78)
Others	81.90 (10)
RMSD	
Bond length (Å)	0.008
Bond angle (°)	0.910
Ramachandran favoured (%)	
Favoured / Allowed / Outliers	97.09 / 2.91 / 0.00
**PDB code**	9G8T

Statistics for the highest-resolution shell are shown in parentheses.

* *R_{free} was calculated using 5% of randomly chosen reflection from each resolution shell.*
